# Dynamics and Control of a Magnetic Transducer Array Using Multi-Physics Models and Artificial Neural Networks

**DOI:** 10.3390/s21206788

**Published:** 2021-10-13

**Authors:** Georgios Tsakyridis, Nikolaos I. Xiros

**Affiliations:** 1 School of Electrical and Computer Engineering, Aristotle University of Thessaloniki, University Campus, 54124 Thessaloniki, Greece; tsakyridis@ece.auth.gr; 2 School of Naval Architecture and Marine Engineering, University of New Orleans, New Orleans, LA 70148, USA

**Keywords:** sensor, magnetic, transducer, control, observer, multi-physics, nonlinear, dynamics, modelling, artificial neural network

## Abstract

A linear mechanical oscillator is non-linearly coupled with an electromagnet and its driving circuit through a magnetic field. The resulting non-linear dynamics are investigated using magnetic circuit approximations without major loss of accuracy and in the interest of brevity. Different computational approaches to simulate the setup in terms of dynamical system response and design parameters optimization are pursued. A current source operating in baseband without modulation directly feeds the electromagnet, which consists commonly of a solenoid and a horseshoe-shaped core. The electromagnet is then magnetically coupled to a mass made of soft magnetic material and attached to a spring with damping. The non-linear system is described by a linearized steady-space representation while is examined for controllability and observability. A controller using a pole placement approach is built to stabilize the element. Drawing upon the fact that coupling works both ways, enabling estimation of the mass position and velocity (state variables) by processing the induced voltage across the electromagnet, a state observer is constructed. Accurate and fast tracking of the state variables, along with the possibility of driving more than one module from the same source using modulation, proves the applicability of the electro-magneto-mechanical transducer for sensor applications. Next, a three-layer feed-forward artificial neural network (ANN) system equivalent was trained using the non-linear plant-linear controller-linear observer configuration. Simulations to investigate the robustness of the system with respect to different equilibrium points and input currents were carried out. The ANN proved robust with respect to position accuracy.

## 1. Introduction

**Figure 1:**
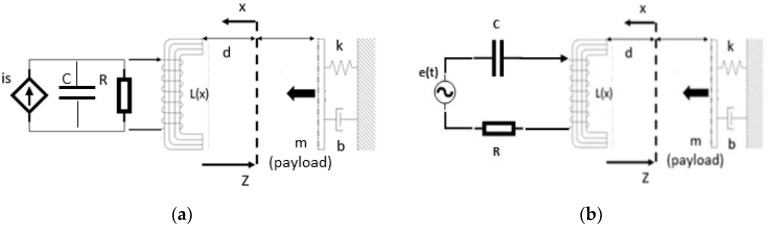
(**a**) Coupled electromechanical oscillator investigated in this work; (**b**) coupled electromechanical oscillator as described in [25].

Advancements in the field of magnetic materials in terms of better efficiency and energy densities [1], together with the integration of mechanics, electromagnetics, power and control electronics into the system, have enabled intelligent electromagnetic actuators and sensors. Electromechanical devices are fundamentally based on quasi-static magnetic and electric fields, where force is generated [2]. Widely spread nowadays due to their simple design and system integration, they can measure changes in the magnetic reluctance of the circuit, which can be correspondingly related to a change in speed or position [3]. Precise operation can be realized using control techniques such as feedback control, which enables them both as sensors and actuators [4]. Typical applications can be found in aerospace [5], automotive [6], ocean [7] and biomedical devices [8] as well as flywheel and transmission speed [9], crank and cam shaft position for engine timing, throttle valve position for air intake, steering wheel position, pedal position, fluid level, chassis height, and in electronic door locks [6]. Given the joint sensing-actuation functionality, electro-magneto-mechanical devices are a promising solution for multi-agent architectures. Such architectures have a vast application field, such as mechatronics and robotics [10], smart grids [11] as well as sensor networks [12]. An agent is a software-controlled hardware, which reacts to a stimulus according to a user-defined behavior [13,14,15,16]. A multi-agent constellation or swarm uses a collaborative approach to achieve a common purpose [17,18,19,20,21,22,23], difficult or impossible to solve by a monolithic system. In this work, we examine a sensor application of an electromechanical oscillator as proposed by Xiros [24,25] (Figure 1). Initially, a force imposed on the mechanical system by the electromagnet induces a displacement of the mass, altering the air gap length. In the next instance, the acting force has changed and the mass moves again. This iterative sequence repeats, forming a closed loop between the displacement and the magnetic force. The magnetically actuated mass-spring-damper system is described and simulated in Section 2. Different simulation methods are presented and compared. Section 3 shows the state-space formulation of the problem together with its linearized form. Using the eigenvalues concept, the system’s’ stability is assessed. In Section 4, closed-loop simulations are conducted to achieve a higher degree of stability. In order to estimate the position and velocity of the oscillating mass, the system states must be known at all times. By designing a state observer for the electromechanical oscillator, position and velocity tracking is achieved. Last, the overall system is replaced by an artificial neural network (ANN) equivalent. The ANN is trained using data collected from the analytical model for different equilibrium points. The current input is kept variable yet close to the equilibrium values where the linearization is still valid.

## 2. The Electromechanical Oscillator 

### 2.1. Lagrangian Formulation of the System

Figure 1a,b show the electromechanical oscillator investigated in this work and in [25] respectively. 

Analysis of dynamic systems requires the equations of motion for such systems to be derived. The ability of computation and analytical approaches depends on the mathematical description used to formulate system dynamics. Often Newtonian mechanics is replaced by Lagrangian dynamics, which solely depends on the energy balance within a non-dissipative system:

(1) ℒ=K−T

Lagrange’s equation can be augmented, using the so-called “power” function, to include dissipative forces as well as other non-dissipative, non-conservative external forces:

(2) $$\frac{d}{dt}\left(\frac{\partial ℒ}{\partial \dot{q_{i}}}\right)-\frac{\partial ℒ}{\partial q_{i}}=\frac{\partial P}{\partial \dot{q_{i}}}$$

Let us now consider the electromechanical system shown in Figure 1a. In specific, the system’s circuit consists of an electric current source, a conductance, a capacitor and the electromagnet’s coil all connected in parallel. The Lagrangian of the system can then be written as:

(3) $$ℒ=K_{e}+K_{m}-(T_{e}+T_{m}),$$

(4) $$K_{e}=\frac{1}{2}CV^{2}=\frac{1}{2}C{\dot{\psi }}^{2}$$

(5) $$T_{e}=\frac{1}{2}L\left(x\right)i_{L}{}^{2}-i_{s}\psi =\frac{1}{2L\left(x\right)}\psi^{2}-i_{s}\psi$$

(6) $$K_{m}=\frac{1}{2}m{\dot{x}}^{2},$$

(7) $$T_{m}=\frac{1}{2}kx^{2}-xf\left(t\right)$$

(8) $$P_{e}=-\frac{{\dot{\psi }}^{2}}{2R}\;,P_{m}=-\frac{{\dot{bx}}^{2}}{2}$$

By substituting (4)–(8) into (3):

(9) $$ℒ=\frac{1}{2}C{\dot{\psi }}^{2}+\frac{1}{2}m{\dot{x}}^{2}-\left(\frac{1}{2L\left(x\right)}\psi^{2}-i_{s}\psi +\frac{1}{2}kx^{2}-xf\left(t\right)\right)$$

For the mechanical subsystem, the Lagrange equation can be written as:

(10) $$\begin{array}{c} \frac{d}{dt}\left(m\dot{x}\right)+\frac{\partial \left(\frac{1}{2}kx^{2}-xf\left(t\right)+\frac{1}{2L\left(x\right)}\psi^{2}\right)}{\partial x}=-b\dot{x}\to m\ddot{x}+kx-f\left(t\right)+\frac{\psi^{2}}{2}\frac{\partial \left(L{\left(x\right)}^{-1}\right)}{\partial x}=-b\dot{x}\to \\ m\ddot{x}+kx+b\dot{x}-\frac{\psi^{2}}{2L^{2}\left(x\right)}\frac{\partial \left(L\left(x\right)\right)}{\partial x}=f\left(t\right)\to m\ddot{x}+kx+b\dot{x}=f\left(t\right)+\frac{\psi^{2}}{2L^{2}\left(x\right)}\frac{\partial \left(L\left(x\right)\right)}{\partial x} \end{array}$$

From the right-hand side of the above, the electromagnetic force applied to the mass by the electromagnet is obtained as follows.

(11) $$F_{em}=\frac{\psi^{2}}{2L^{2}\left(x\right)}\frac{\partial \left(L\left(x\right)\right)}{\partial x}$$

The system has two degrees of freedom, thus for the electromagnetic part:

(12) $$\frac{d}{dt}\left(C\dot{\psi }\right)+\frac{\partial \left(\frac{\psi^{2}}{2L\left(x\right)}-i_{s}\psi \right)}{\partial \psi }=-\frac{\dot{\psi }}{R}\to C\ddot{\psi }+\frac{\dot{\psi }}{R}+\frac{1}{L\left(x\right)}\psi =i_{s}\overset{C=0}{\to }\dot{\frac{\psi }{R}}+\frac{1}{L\left(x\right)}\psi =i_{s}$$

Note that for C=0: $i_{c}=C\frac{dV}{dt}=0$ equivalent to an open circuit.

### 2.2. Electromagnetic Subsystem

In this work the magnetic circuit solution is adopted, based on Maxwell’s equations and the correspondence of the differential equations for linear magnetization problems with those for steady current problems. This is only true in the special case where boundary conditions for the two cases are identical [26]. In order to simplify the analysis of magnetic circuits and make them suitable and accurate enough for engineering applications, assumptions must be made. Initially, the terms of the Maxwell equations containing time-varying electric fields are neglected and the system is regarded as magneto quasi-static. The second assumption refers to the magnetic circuit, which is thought of as a structure of high-permeability materials. This results in the magnetic flux to flow entirely through the path defined by the structure.

**Figure 2:**
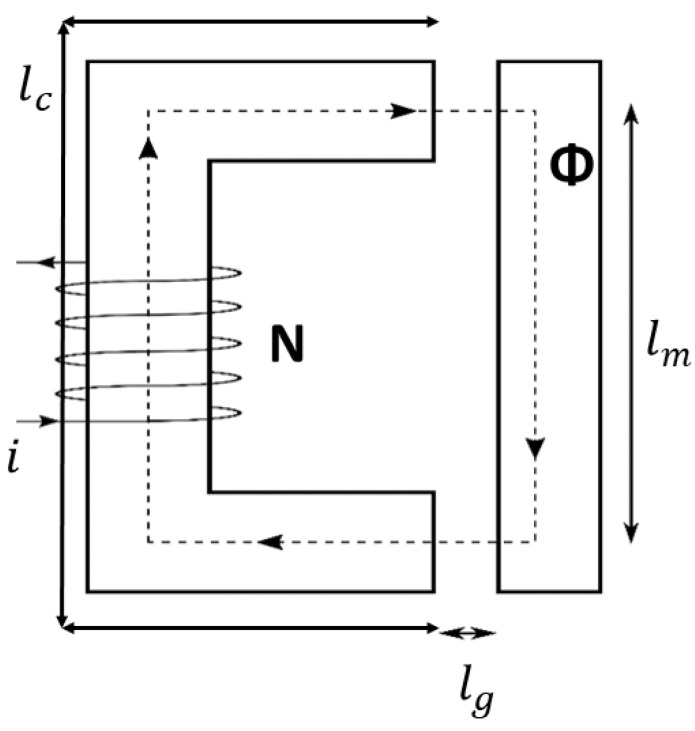
Electromagnet featuring an air gap and a moving mass.

An electromagnet featuring a solenoid around a horseshoe-shaped core, an air gap and a moving mass is shown in Figure 2.

The magnetic reluctances ℜ of the circuit are defined as:

(13) $${ℜ}_{c}=\frac{l_{c}}{A_{c}\mu_{0}\mu_{c}}$$

(14) $${ℜ}_{m}=\frac{l_{m}}{A_{r}\mu_{0}\mu_{m}}$$

(15) $${ℜ}_{g}=\frac{l_{g}}{A_{g}\mu_{0}}$$

where $A_{m}=A_{c}=A_{g}=A$. 

The magnetomotive force (mmf) drives a magnetic flux Φ through the magnetic reluctances of the core, air gap and moving mass. From (13), (14) and (15) it is clear that the magnetic reluctance depends on the material permeability. High magnetic permeability can result in small reluctances, in fact much smaller than that of the air gap. The resulting magnetic field stores energy in the air gap equal to:

(16) $$\;E=\frac{1}{2\mu_{0}}B^{2}A_{g}l_{g}$$

The force in the gap acting on the mass is given by the rate of change of energy with gap length, thus:

(17) $$F_{em}=\frac{dE}{dl_{g}}\;\overset{\left(11\right)}{\to }\;F_{em}=\frac{1}{2\mu_{0}}A_{g}B^{2}$$

It is known that:

(18) $$B=\frac{Ni_{L}}{A\left({ℜ}_{c}+{ℜ}_{m}+2{ℜ}_{g}\right)}\;$$

thus we can write (17) as:

(19) $$F_{em}=\frac{1}{2\mu_{0}}\frac{N^{2}i_{L}{}^{2}}{A_{g}{\left({ℜ}_{c}+{ℜ}_{m}+2{ℜ}_{g}\right)}^{2}}$$

or alternatively:

(20) $$F_{em}=\frac{N^{2}A\mu_{0}\mu_{c}{}^{2}\mu_{m}{}^{2}}{{\left(l_{c}\mu_{m}+\mu_{c}l_{m}+2\mu_{c}\mu_{m}x\left(t\right)\right)}^{2}}i^{2}$$

The force, acceleration, velocity and displacement are vectors. The electromagnetic force is acting on the mass minimizing the gap length.

Let us now define the two constants (assuming $\mu_{c}=\mu_{m}=\mu$):

(21) $$K_{a}=\frac{1}{2}N^{2}A\mu_{0}$$

(22) $$K_{b}=\frac{l_{c}\mu_{m}+l_{m}\mu_{c}}{\mu_{c}\mu_{m}}$$

We can now write (20):

(23) $$F_{em}=\frac{2K_{a}i_{L}{}^{2}}{{\left(K_{b}+2x\right)}^{2}}$$

and (11) as:

(24) $$\ddot{x}=\frac{2K_{a}i_{L}{}^{2}}{m{\left(K_{b}+2x\right)}^{2}}-\frac{Kx}{m}-\frac{b\dot{x}}{m}$$

The dynamic equations for the electrical subsystem must be derived as well. Knowledge of the induced voltage and variable inductance are key for both sensory and actuating applications.

(25) $$V_{L}=\frac{d\left(L\left(x\right)i_{L}\right)}{dt}\underset{}{\to }V_{L}=L\left(x\right)\frac{di_{L}}{dt}+i_{L}\frac{dL\left(x\right)}{dx}\frac{dx}{dt}$$

It is known that:

(26) $$\psi =\frac{Ni_{L}\left(t\right)}{\left(\frac{l_{c}}{A_{c}\mu_{0}\mu_{c}}+\frac{l_{m}}{A_{r}\mu_{0}\mu_{m}}+2\frac{x\left(t\right)}{A_{g}\mu_{0}}\right)}$$

and:

$$L=\frac{N\psi }{i_{L}}\underset{}{\to }$$

(27) $$L=\frac{N^{2}}{\left(\frac{l_{c}}{A_{c}\mu_{0}\mu_{c}}+\frac{l_{m}}{A_{r}\mu_{0}\mu_{m}}+2\frac{x\left(t\right)}{A_{g}\mu_{0}}\right)}=\frac{N^{2}A_{c}\mu_{0}\mu_{c}\mu_{m}}{l_{c}\mu_{m}+\mu_{c}l_{m}+2\mu_{c}\mu_{m}x\left(t\right)}$$

We assume:

(28) $$\xi_{0}=N^{2}A_{c}\mu_{0}\mu_{c}\mu_{m},\;\;\xi_{1}=l_{c}\mu_{m}+\mu_{c}l_{m},\;\;\xi_{2}=2\mu_{c}\mu_{m}$$

and (27) can now be written as:

(29) $$L\left(x\right)=\frac{\xi_{0}}{\xi_{1}+\xi_{2}x\left(t\right)}$$

### 2.3. Electromechanical System Simulation

The dynamical electromechanical system described in this work is simulated using Matlab [27], in particular:

- Matlab Simulink Simscape;
- Matlab Simulink;

#### 2.3.1. Simscape Implementation

**Figure 3:**
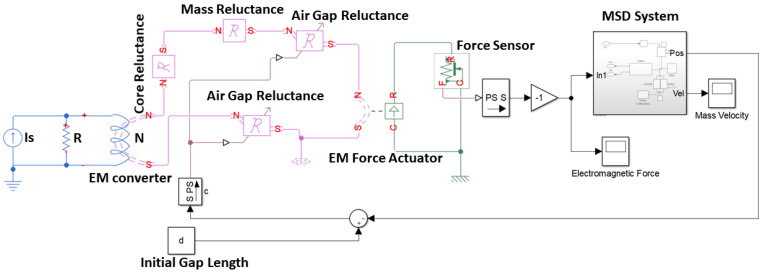
Simulink Simscape implementation of the electromechanical oscillator using the analytical expressions.

**Figure 4:**
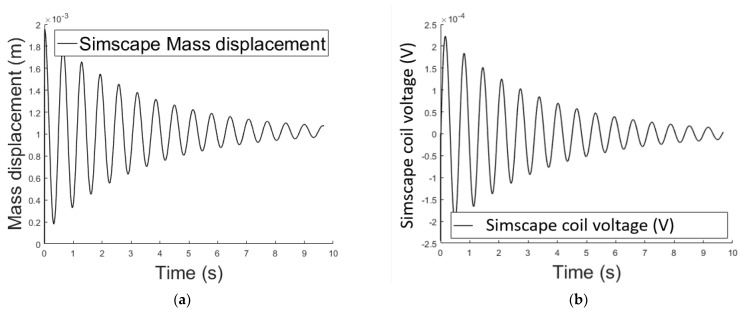
(**a**) Air gap length variation over time using the Simscape implementation; (**b**) variable voltage across the electromagnet’s coil over time using the Simscape implementation.

Matlab Simscape uses physical blocks to model complex systems (Figure 3). Each block models an item which is interconnected with others, forming a diagram equivalent to the mathematical model of the system. The interaction of the blocks is accomplished through signal ports permitting non-directional energy exchange. Additional signal ports are available to the blocks in order to specify initial conditions and enhance the model’s computational speed. Blocks are available for both active and passive elements depending on their ability to deliver energy to the system, store it, or dissipate it. Results of the simulated system are shown in Figure 4.

#### 2.3.2. Simulink Implementation

Matlab Simulink allows dynamical modelling using a graphic environment throughout signal blocks. These blocks are complete only when input and output signals are fully defined. The magnetic force function is built and integrated into the Simulink model using the approximation described in (29) for the inductance.

**Figure 5:**
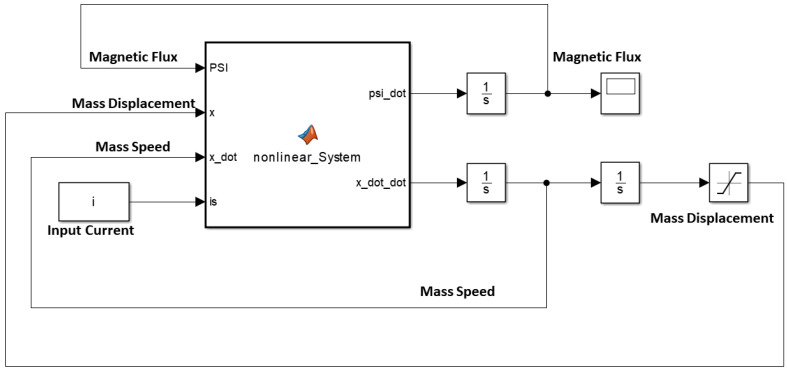
Simulink implementation of the electromechanical oscillator.

**Figure 6:**
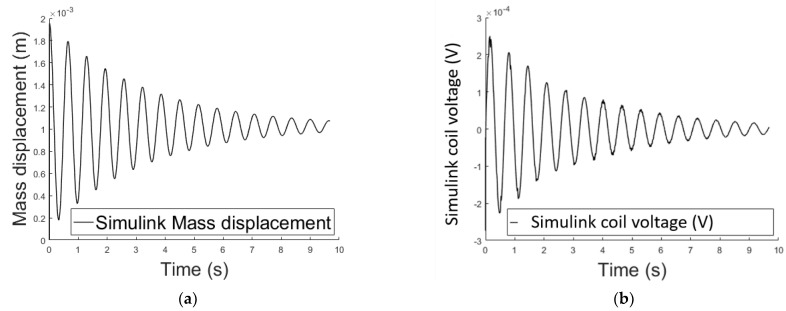
(**a**) Air gap length variation over time using the analytical Simulink implementation; (**b**) variable voltage across the electromagnet’s coil over time using the analytical Simulink implementation.

Figure 5 and Figure 6 shows the air gap length and the Simulink coil voltage over a simulation time of 10 s, for the implementation in Figure 5. Initially the output voltage is equal to zero, since the system is in equilibrium (no spring deformation). When a current i is applied to the circuit an attractive force is pulling the mass toward the electromagnet (Figure 2), changing the air gap length thus inducing voltage on the inductance. When the spring force overcomes the magnetic force the mass moves away from the electromagnet. Motion on the positive x direction induces a positive voltage (negative x direction motion results in negative induced voltage).

#### 2.3.3. Methods Comparison

**Figure 7:**
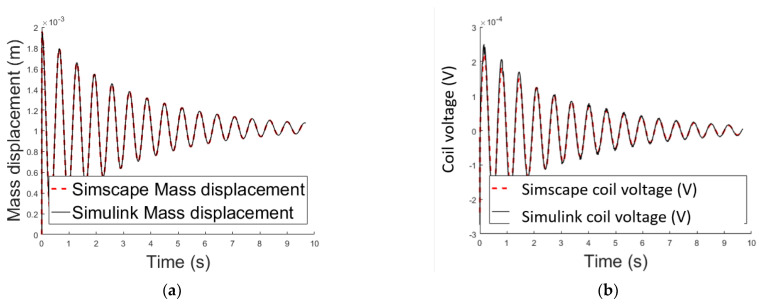
(**a**) Air gap length variation over time methods comparison; (**b**) variable voltage across the electromagnet’s coil over time methods comparison.

Next the two methods described above to perform the dynamic simulation of the system are compared. Figure 7 confirms that the results are comparable hence both methods are suitable.

## 3. State-Space Problem Formulation

The state-space model is defined in terms of the derivatives of the states and its representation is given below:

$$\dot{x}=Ax+Bu$$

(30) y=Cx+Du

In order to formulate the state equations of the electro-magneto-mechanical oscillator shown in Figure 1, constraints limiting the system according to its physical and performance characteristics must be defined. For the inductance, we assume the expression described by (29), derived using the magnetic circuit approximation. In favor of simplicity:

(31) $$L=\frac{\xi_{0}}{\xi_{1}+\xi_{2}x\left(t\right)}$$

where

(32) $$\xi_{0}=N^{2}A_{c}\mu_{0}\mu_{c}\mu_{m}$$

(33) $$\xi_{1}=l_{c}\mu_{m}+\mu_{c}l_{m}$$

(34) $$\xi_{2}=2\mu_{c}\mu_{m}$$

Figure 1 shows the origin of the mechanical coordinate (*x*), assumed equivalent to the neutral position of the spring while that of the electromagnetic system (*z*) at the position where the mass is marginally in contact with the magnet armature, such that:

(35) z=d−x [m]

thus:

(36) −d<x<d [m]

Based on the expression derived for the electromagnetic force earlier where the magnetic flux and consequently the current appears squared, one can easily derive that the force will be unidirectional, and always attractive, regardless of the electric current’s sign:

(37) $$F_{em}\geq 0\;\left[N\right]$$

Additionally, given that we want to avoid any physical contact in the system, the velocity of a moving part must be controlled and constrained as follows [28]:

(38) $$-s\left(d-x\right)<\dot{x}<s\left(d-x\right)\;\left[m/s\right]$$

where *s* is a constant such that $\dot{x}\in \left[-5,5\right]\;\left[m/s\right]$.

According to Kirchhoff’s law, Newton’s second law and the Lagrangian dynamics analysis presented earlier, with the electromagnetic force acting on the moving mass the state equations are:

(39) $$\begin{array}{c} \dot{\psi }=Ri_{s}-\frac{R}{L\left(x\right)}\psi \\ \dot{\psi }=Ri_{s}-\frac{R\xi_{0}}{\xi_{1}+\xi_{2}x\left(t\right)}\psi =𝒽\left(x,\dot{x},\psi \right)\; \end{array}$$

(40) $$\ddot{x}=\frac{\;\xi_{2}\psi^{2}\;}{2m\xi_{0}}-\frac{k}{m}x-\frac{b}{m}\dot{x}=𝒻\left(x,\dot{x},\psi \right)$$

with states: the mass potion x, the mass velocity $\dot{x}$ and the magnetic flux ψ.

Assuming the voltage across the inductance to be the systems’ output:

(41) $$V_{L}=\dot{\psi }=Ri_{s}-\frac{R\xi_{0}}{\xi_{1}+\xi_{2}x\left(t\right)}\psi =𝒽\left(x,\dot{x},\psi \right)$$

(39)–(41) represent the dynamics of a nonlinear system thus linearization is applied around some equilibrium point $\left(x_{0},{\dot{x}}_{0},\psi_{0},i_{s0}\right)$ using Taylor expansion:

(42) $$\begin{array}{c} 𝓉\left(x,\dot{x},\psi \right)=𝓉\left(x_{0},{\dot{x}}_{0},\psi_{0},i_{s0}\right)+{𝓉}_{x}^{\prime }\left(x_{0},{\dot{x}}_{0},\psi_{0},i_{s0}\right)\left(x-x_{0}\right)+{𝓉}_{\dot{x}}^{\prime }\left(x_{0},{\dot{x}}_{0},\psi_{0},i_{s0}\right)\left(\dot{x}-{\dot{x}}_{0}\right)+\\ {𝓉}_{\psi }^{\prime }\left(x_{0},{\dot{x}}_{0},\psi_{0},i_{s0}\right)\left(\psi -\psi_{0}\right)+{𝓉}_{\psi }^{\prime }\left(x_{0},{\dot{x}}_{0},\psi_{0},i_{s0}\right)\left(i_{s}-i_{s0}\right)\;, \end{array}$$

where:

(43) $${𝓉}_{x}^{\prime }\left(x_{0},{\dot{x}}_{0},\psi_{0},i_{s0}\right)=\frac{\partial 𝒽}{\partial x}\left(x_{0},{\dot{x}}_{0},\psi_{0},i_{s0}\right)\;or\;\frac{\partial 𝒻}{\partial x}\left(x_{0},{\dot{x}}_{0},\psi_{0},i_{s0}\right)\;\;$$

(44) $${𝓉}_{\dot{x}}^{\prime }\left(x_{0},{\dot{x}}_{0},\psi_{0},i_{s0}\right)=\frac{\partial 𝒽}{\partial \dot{x}}\left(x_{0},{\dot{x}}_{0},\psi_{0},i_{s0}\right)\;or\;\frac{\partial 𝒻}{\partial \dot{x}}\left(x_{0},{\dot{x}}_{0},\psi_{0},i_{s0}\right)\;$$

(45) $${𝓉}_{\psi }^{\prime }\left(x_{0},{\dot{x}}_{0},\psi_{0},i_{s0}\right)=\frac{\partial 𝒽}{\partial \psi }\left(x_{0},{\dot{x}}_{0},\psi_{0},i_{s0}\right)\;or\;\frac{\partial 𝒻}{\partial \psi }\left(x_{0},{\dot{x}}_{0},\psi_{0},i_{s0}\right)\;\;$$

and for the equilibrium point ${\dot{x}}_{0}=0,\;{\ddot{x}}_{0}=0$ and,

(46) $$\psi_{0}=\sqrt{\frac{2\xi_{0}Kx_{0}}{\xi_{2}}}$$

(47) $$i_{s0}=\frac{\psi_{0}}{L\left(x_{0}\right)}$$

By substituting (39)–(41) into (43)–(45):

(48) $${𝒽}_{x}^{\prime }\left(x_{0},{\dot{x}}_{0},\psi_{0},i_{s0}\right)=-R\psi_{0}\frac{\xi_{2}}{\xi_{0}}\;$$

(49) $${𝒽}_{\dot{x}}^{\prime }\left(x_{0},{\dot{x}}_{0},\psi_{0},i_{s0}\right)=0$$

(50) $${𝒽}_{\psi }^{\prime }\left(x_{0},{\dot{x}}_{0},\psi_{0},i_{s0}\right)=-\frac{R\left(\xi_{1}+\xi_{2}x_{0}\right)}{\xi_{0}}$$

(51) $${𝒻}_{x}^{\prime }\left(x_{0},{\dot{x}}_{0},\psi_{0},i_{s0}\right)=-\frac{k}{m}$$

(52) $${𝒻}_{\dot{x}}^{\prime }\left(x_{0},{\dot{x}}_{0},\psi_{0},i_{s0}\right)=-\frac{b}{m}$$

(53) $${𝒻}_{\psi }^{\prime }\left(x_{0},{\dot{x}}_{0},\psi_{0},i_{s0}\right)=\frac{\;2\xi_{2}\;\psi_{0}}{m\xi_{0}}$$

and (39)–(41) are written as:

(54) $$\dot{\psi }=\left(-R\psi_{0}\frac{\xi_{2}}{\xi_{0}}\right)\left(x-x_{0}\right)-\frac{R\left(\xi_{1}+\xi_{2}x_{0}\right)}{\xi_{0}}\left(\psi -\psi_{0}\right)+R\left(i_{s}-i_{s0}\right)\;$$

(55) $$\ddot{x}=-\frac{k}{m}\left(x-x_{0}\right)-\frac{b}{m}\left(\dot{x}-{\dot{x}}_{0}\right)+\frac{\;2\xi_{2}\;\psi_{0}}{m\xi_{0}}\left(\psi -\psi_{0}\right)$$

(56) $$V_{L}=\left(-R\psi_{0}\frac{\xi_{2}}{\xi_{0}}\right)\left(x-x_{0}\right)-\frac{R\left(\xi_{1}+\xi_{2}x_{0}\right)}{\xi_{0}}\left(\psi -\psi_{0}\right)+R\left(i_{s}-i_{s0}\right)$$

We define as state variables the displacement x, the velocity $\dot{x}$, the magnetic flux ψ and adopt the variable transformation $\delta x=x-x_{0}$ $\dot{\delta x}=\dot{x}-\dot{x_{0}}$, $\delta \psi =\psi -\psi_{0}$ and $\delta i=i_{s}-i_{s0}$. (30) is now written as:

(57) $$\left[\begin{array}{c} \delta \dot{x}\\ \delta \ddot{x}\\ \delta \dot{\psi } \end{array}\right]=\left[\begin{array}{ccc} 0 & 1 & 0\\ -\frac{k}{m} & -\frac{b}{m} & \frac{\;2\xi_{2}\;\psi_{0}}{mL_{0}}\\ -R\psi_{0}\frac{\xi_{2}}{\xi_{0}} & 0 & -\frac{R\left(\xi_{1}+\xi_{2}x_{0}\right)}{\xi_{0}} \end{array}\right]\left[\begin{array}{c} \delta x\\ \dot{\begin{array}{c} \delta x\\ \delta \psi \end{array}} \end{array}\right]+\left[\begin{array}{c} 0\\ 0\\ R \end{array}\right]\left[\delta i\right]$$

(58) $$y=\left[\left(-R\psi_{0}\frac{\xi_{2}}{\xi_{0}}\right)\;\;\;0\;-\frac{R\left(\xi_{1}+\xi_{2}x_{0}\right)}{\xi_{0}}\right]\left[\begin{array}{c} \delta x\\ \dot{\begin{array}{c} \delta x\\ \delta \psi \end{array}} \end{array}\right]+\left[\begin{array}{c} 0\\ 0\\ R \end{array}\right]\left[\delta i\right]$$

and:

(59) $$A=\left[\begin{array}{ccc} 0 & 1 & 0\\ \left(a^{2}\xi_{1}e^{-a\left(d-x_{0}\right)}-\frac{2a^{2}\xi^{2}{}_{1}e^{-2a\left(d-x_{0}\right)}}{L\left(x_{0}\right)}\right)\frac{\psi_{0}{}^{2}}{m}-\frac{k}{m} & -\frac{b}{m} & \frac{\;2a\xi_{1}e^{-a\left(d-x_{0}\right)}\;\psi_{0}}{mL{\left(x_{0}\right)}^{2}}\\ \left(\frac{Ra\xi_{1}e^{-a\left(d-x\right)}}{L{\left(x_{0}\right)}^{2}}\psi_{0}\right) & 0 & -\frac{R}{L\left(x_{0}\right)} \end{array}\right]$$

(60) $$B=\left[\begin{array}{c} 0\\ 0\\ R \end{array}\right]$$

(61) $$C=\left[\left(-R\psi_{0}\frac{\xi_{2}}{\xi_{0}}\right)\;\;\;0\;-\frac{R\left(\xi_{1}+\xi_{2}x_{0}\right)}{\xi_{0}}\right]$$

(62) D=[R]

The partial derivatives described in (43)–(45) to linearize the system are calculated using both the analytical and numerical methods around an equilibrium point point $x_{0}=0.015\;m$ (Table 1).

and:

(63) $$A=\left[\begin{array}{ccc} 0 & 1 & 0\\ -100 & -0.06 & -910.45\\ -124.86 & 0 & -390.76 \end{array}\right]$$

(64) $$B=\left[\begin{array}{c} 0\\ 0\\ 2 \end{array}\right]$$

The system output is the voltage across the inductance in equilibrium position, thus (51):

(65) C=[−124.86 0−390.76]

(66) D=[2]

Table 2 summarizes the state variables, inputs and outputs of the linearized system:

Let us now compute the eigenvalues of A to determine whether the linearized open-loop system (without feedback control) is stable. The poles of the transfer function are the solution to:

(67) det(sI−A)=0

thus: $p_{1}=-910.36$, $p_{2}=-1.05+3.64i$ and $p_{3}=-1.05+3.64i$. All of three poles lie in the left-half plane making the system stable.

## 4. System Controllability and Observability

A linear time invariant system $\dot{x}=Ax+Bu,\;x\left(0\right)=x_{0}$ is controllable if over the interval $\left[0,t_{f}\right]$ a control input $u\left(t\right)\;\forall \;t\in \left[0,t_{f}\right]$ steers the state from $x_{init}$ to $x_{t_{f}}$ according to:

(68) $$x\left(t_{f}\right)=e^{At_{f}}x_{init}+\int_{0}^{t_{f}}e^{A\left(t-\tau \right)}Bu\left(\tau \right)d\tau$$

or in other words an LTI system is controllable at time $t_{f}>0$ if for any initial state and for any target state $x_{t_{f}}$, a control input u(t) exists that can steer the system from x(0) to $x_{t_{f}}$ over the defined interval.

It can be shown that an LTI system is controllable if and only if its controllability matrix M has full rank, where M=[B, AB]. For our system, from (63) and (64):

(69) $$M=\left[\begin{array}{ccc} 0 & 0 & 1.2486\;\times \;10^{3}\\ 0 & 1.2486\;\times \;10^{3} & -1.1393\;\times \;10^{6}\\ 2 & -1.8209\;\times \;10^{3} & 1.6578\;\times \;10^{6} \end{array}\right]$$

The controllability matric M has full rank since rank (M) is equal to number of state variables.

An LTI system is observable at time $t_{f}$ if the initial state x(0) can be uniquely determined from any given $u\left(0\right),\;\ldots ,\;u\left(t_{f}-1\right),\;y\left(0\right),\;\ldots ,y\left(t_{f}-1\right)$, where y is the output of the system. By analogy, an LTI system is observable if and only if its observability matrix O has full rank, where O=[C, AC]. For our system, from (63) and (65):

(70) $$O=\left[\begin{array}{ccc} 0 & 0 & 1\\ 124.8627 & 0 & -910.4529\\ -1.1368\;\times \;10^{5} & 124.8627 & 8.2892\;\times \;10^{5} \end{array}\right]$$

The observability matrix O has full rank since rank (O) is equal to number of state variables.

Let’s now consider the linearized system as described in Section 3 with state variables the spring displacement x and the velocity $\dot{x}$, and output the inductance voltage $V_{L}$. As stated earlier, the system contains an unstable pole with a corresponding damping ratio of −1. A negative damping ratio, called driving force, increases the system oscillation rather than driving the system to stability (damping force).

### 4.1. Linear Controller Design Using Pole Placement

**Figure 8:**
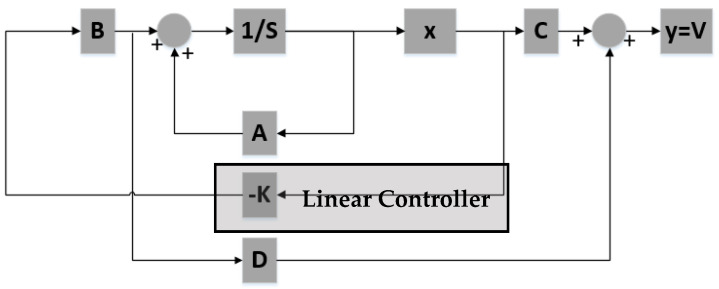
Full-state feedback using poles placement block diagram.

To stabilize the system around a specific position, a feedback controller is designed using poles placement (Figure 8). In order to implement full-state feedback all state variables must be known to the controller at all times.

The controller equation is:

(71) $$u=-K_{gain}x$$

where u is the system input, $K_{gain}$ the negative state-feedback gain matrix and x the state variables, and:

(72) $$\dot{x}=Ax+B\left(-K_{gain}x\right)=\left(A-BK_{gain}\right)x$$

(73) y=Cx+Du

where:

(74) $$A=\left[\begin{array}{ccc} 0 & 1 & 0\\ -100 & -2 & 624.3134\\ 124.86 & 0 & -910.4529 \end{array}\right]$$

(75) $$B=\left[\begin{array}{c} 0\\ 0\\ 2 \end{array}\right]$$

(76) C=[−124.86 0−390.76]

(77) D=2

The system specific closed loop $A_{cl}$ matrix is now equal to $\left(A-BK_{gain}\right)$ and the gain $K_{gain}$ matrix is a 1 × 2 matrix hence,

(78) $$A_{cl}=\left[\begin{array}{ccc} 0 & 1 & 0\\ -100 & -2 & 624.3134\\ 124.86 & 0 & -910.4529 \end{array}\right]-\left[\begin{array}{c} 0\\ 0\\ 2 \end{array}\right]\left[K_{gain1}\;K_{gain2}\;K_{gain3}\right]$$

The eigenvalues of $A_{cl}$ are given by $\det(A_{cl}-\lambda I)$:

(79) $$\det(A_{cl}-\lambda I)=0$$

Let us now assume that we would like to place two poles $p_{1}$ and $p_{2}$ such that the characteristic equation is:

(80) $$\left(\lambda +p_{1}\right)\left(\lambda +p_{2}\right)\left(\lambda +p_{3}\right)=0$$

The gain matrix $K_{gain}$ can now be calculated by setting the coefficients equal to each other.

The location of the poles $p_{1}$ and $p_{2}$ characterize the stability and time-domain performance of the system.

The controller design and the selection of the gain matrix $K_{gain}$ are based on two criteria:

1. overshot <5%
2. settling time <0.5 sec

For an overshoot less than 5% we have:

(81) $$\begin{array}{cc} \exp\left(-\frac{\zeta \pi }{\sqrt{1-\zeta^{2}}}\right) & <0.05\to -\frac{\zeta \pi }{\sqrt{1-\zeta^{2}}}<\ln\left(0.05\right)=-3.00\to \frac{\zeta }{\sqrt{1-\zeta^{2}}}>0.95\\ & \to \frac{\zeta^{2}}{1-\zeta^{2}}>0.91\to \zeta^{2}>\frac{0.91}{1+0.91}\to \zeta^{2}>0.48\to \zeta^{2}>0.69\; \end{array}$$

and for a settling time (3% error) of less than 0.5 s:

(82) $$t_{s}=\frac{\ln\left(0.03\right)}{\zeta \omega }<0.5\to \zeta \omega >7\;$$

For a second order system with ζ<1 the poles are given by:

(83) $$-\zeta \omega \mp i\omega \sqrt{1-\zeta^{2}}\;$$

The closed loop system showed in Figure 8 is simulated using the following three alternative pole sets:

- $p_{11}=-10+i10$, $p_{12}=-10-i10$ and $p_{13}=-50$
- $p_{21}=-20+i20$, $p_{22}=-20-i20$ and $p_{23}=-100$
- $p_{31}=-30+i30$, $p_{32}=-30-i30$ and $p_{33}=-150$

with gain matrixes:

- $K_{gain1}=\left[K_{gain11}\;K_{gain12}\;K_{gain13}\right]=\left[64.99\;0.772-421.22\right]$
- $K_{gain2}=\left[K_{gain21}\;K_{gain22}\;K_{gain23}\right]=\left[115.44\;3.54-386.22\right]$
- $K_{gain3}=\left[K_{gain31}\;K_{gain32}\;K_{gain33}\right]=\left[262.01\;8.23-351.22\right]$

respectively.

**Figure 9:**
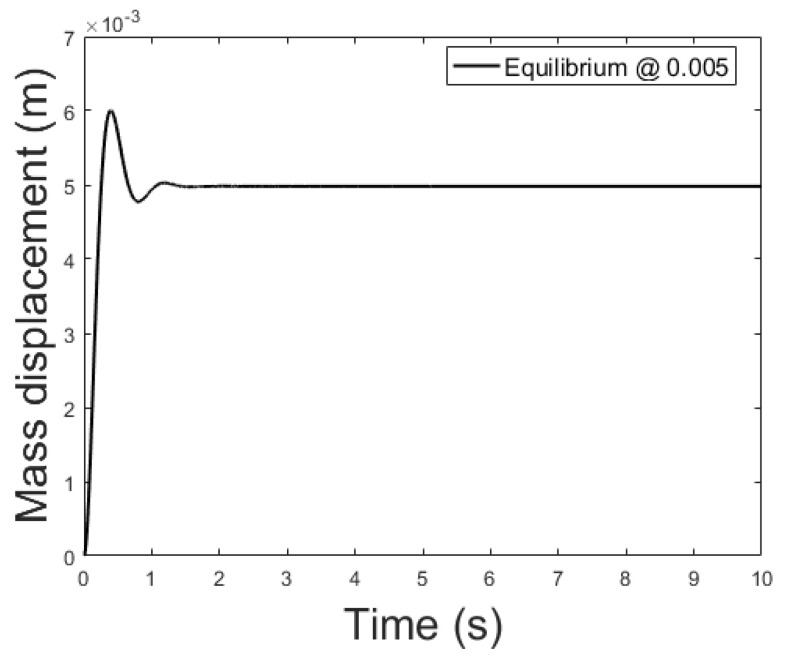
Controlled system output with initial conditions: $x_{0}=0$ m, $\dot{x_{0}}=0$ m/s, ψ=0 Wb and equilibrium at *x* = 0.005.

**Figure 10:**
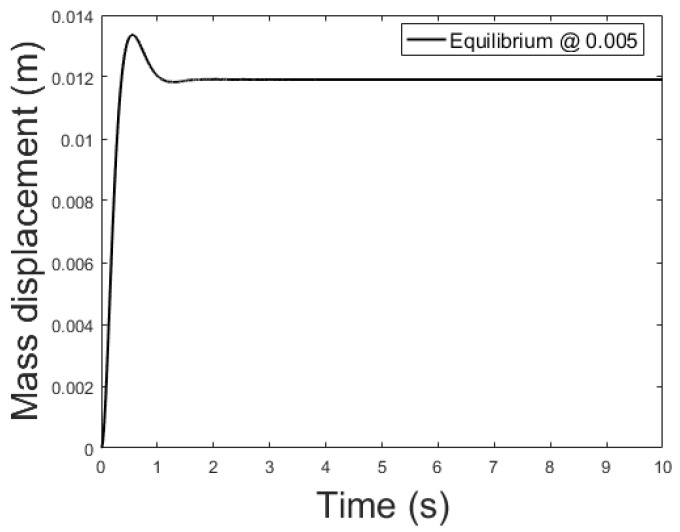
Controlled system output with initial conditions: $x_{0}=0$ m, $\dot{x_{0}}=0$ m/s, ψ=0 Wb and equilibrium at *x* = 0.01.

**Figure 11:**
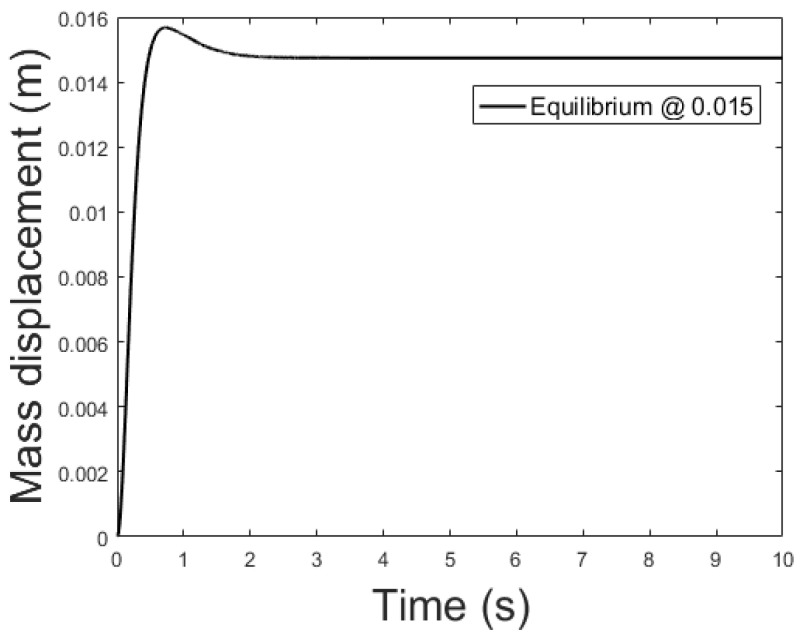
Controlled system output with initial conditions: $x_{0}=0$ m, $\dot{x_{0}}=0$ m/s, ψ=0 Wb and equilibrium at *x* = 0.015.

The controller is based on the linearized system while the plant is the non-linear system described by (39)–(41). The current applied to the plant features a small delta with respect to the equilibrium current, $\delta i_{s0}=0.01\;A$, to remain in the region where the linearization is valid. Figure 9, Figure 10 and Figure 11 show the controlled mass displacement for different equilibrium points using poles set as $p_{31}$, $p_{32}$ and $p_{33}$.

### 4.2. Observer Design

The pole placement method described earlier requires knowledge of the system states at all times. In practice, this is very demanding due to unavailable or expensive sensors often driven by high noise as well. Moreover, some types of application require the system to operate as a sensor rather than as an actuator. Given the proven observability of the studied system, a state observer is designed to overcome the issue. A state observer is capable of estimating and observing the system states regardless of whether some state variables are available for direct measurement or not. The only measurable output for the hereby-described electromechanical oscillator is the inductance voltage. The observer mimics the model of the plant, except for an additional term, introduced to compensate for inaccuracies in the matrices A and B. This new term, called estimation error, is equal to the difference between the estimated output value and the measured value.

The observer equations are:

(84) $$\begin{array}{c} \hat{\dot{x}}=A\hat{x}+Bu+L\left(y-\hat{y}\right)=A\hat{x}+Bu+L\left(y-\hat{Cx}-Du\right)\\ =\left(A-LC\right)\hat{x}+\left(B-LD\right)u+Ly \end{array}$$

(85) $$\hat{y}=C\hat{x}-Du$$

where $\hat{x}$ the estimated states, $\hat{y}$ the estimated output and L the observer gain matrix.

The system’s output is compared to the estimated output. The resulting error is corrected to help the observer converge to the system state values. The observer’s error dynamic is given by:

(86) $$\dot{e}=\dot{x}-\hat{\dot{x}}=\left(A-LC\right)e$$

The input to the open-loop plant is equal to:

(87) $$u=-K_{gain}\hat{x}$$

Thus:

(88) $$\begin{array}{c} \dot{x}=Ax+Bu=Ax+B\left(-K_{gain}x\right)=\left(A-BK_{gain}\right)x-BK_{gain}\left(\hat{x}-x\right)\overset{}{\Rightarrow }\;\dot{x}\\ =\left(A-BK_{gain}\right)x+BK_{gain}e \end{array}$$

and:

(89) $$\left[\begin{array}{c} \dot{x}\\ \dot{e} \end{array}\right]=\left[\begin{array}{cc} A-BK_{gain} & BK_{gain}\\ 0 & \left(A-LC\right) \end{array}\right]\left[\begin{array}{c} x\\ e \end{array}\right]$$

(88) shows that the state dynamics is decoupled from the observer’s error *e* (separation principle) thus the gain K can be computed independently of the observe gain L. The gain matrix $K_{gain}$ is computed, for $p_{31}=-30+i30$, $p_{32}=-30-i30$, $p_{33}=-150$ and is equal to $K_{gain}=\left[262.01\;8.23-351.22\right]$. For the observer, we select eigenvalues located at least five times further left on the complex plane than the plant eigenvalues, since we want the dynamics of the observer to be much faster than the system itself. In other words, the observer must converge to the plants’ states before they converge to zero. Hence we choose:$\;p_{obs1}=-150+6i$, $p_{obs2}=-150-6i$ and $p_{obs3}=-750$. Poles placed further apart than that could result in complex systems with large bandwidth, making the system vulnerable to noise effects. The analytical procedure to derive the observer matrix is identical to the one described for the gain matrix $K_{gain}$. Alternatively, one can use the place command in Matlab and: $L=\left[-9.405\;\times \;10^{3}\;1.914\;\times \;10^{3}-1.29\;3.23\;\times \;10^{3}\right]$. The selection of matrix L is a tradeoff between a fast dynamic response and noise reduction.

**Figure 12:**
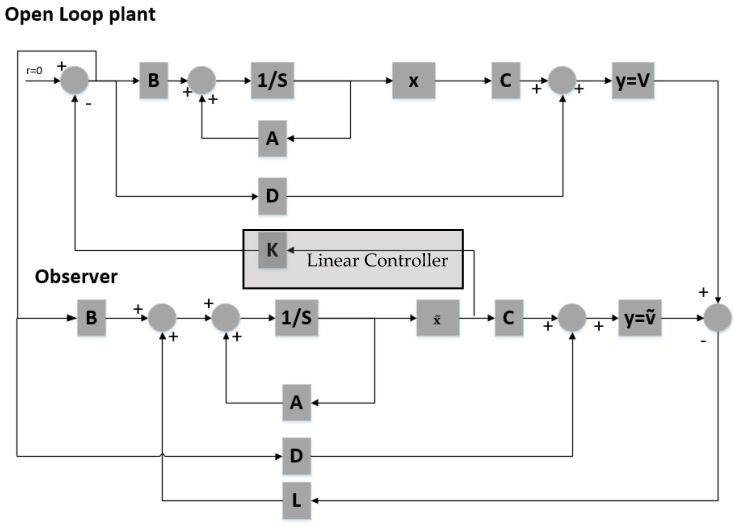
Closed loop block diagram.

**Figure 13:**
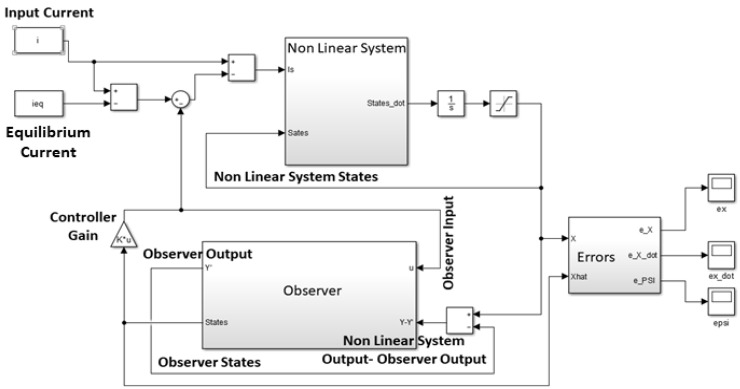
Simulink implementation of the nonlinear system, controller and observer.

Figure 12 shows the non-linear plant-linear controller-linear observer block diagram. Figure 13 shows theMatlab Simulink system implementation. The states are now unknown to the controller, hence the observer is used to generate estimates by comparing the plant output to the observer output. These estimates of the states are fed into the linear controller to stabilize the system around an equilibrium point.

**Figure 14:**
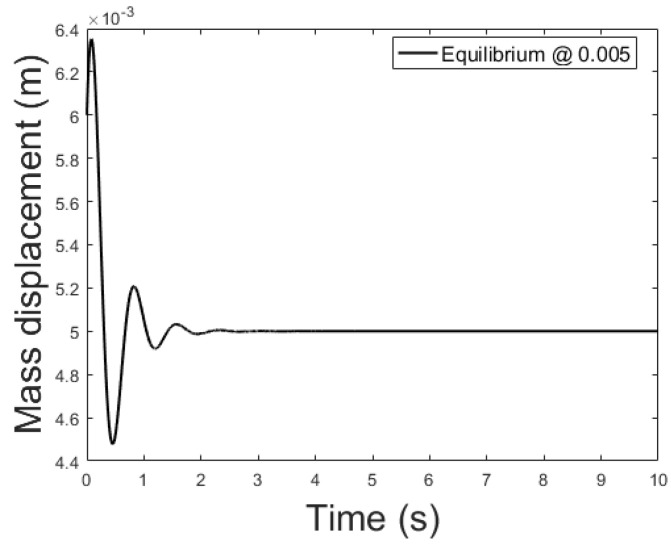
Observer controlled system output with equilibrium at *x* = 0.005.

**Figure 15:**
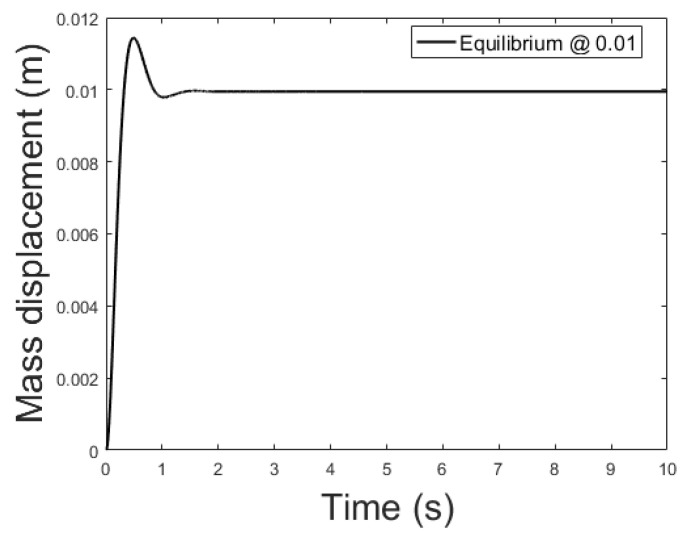
Observer controlled system output with equilibrium at *x* = 0.010.

**Figure 16:**
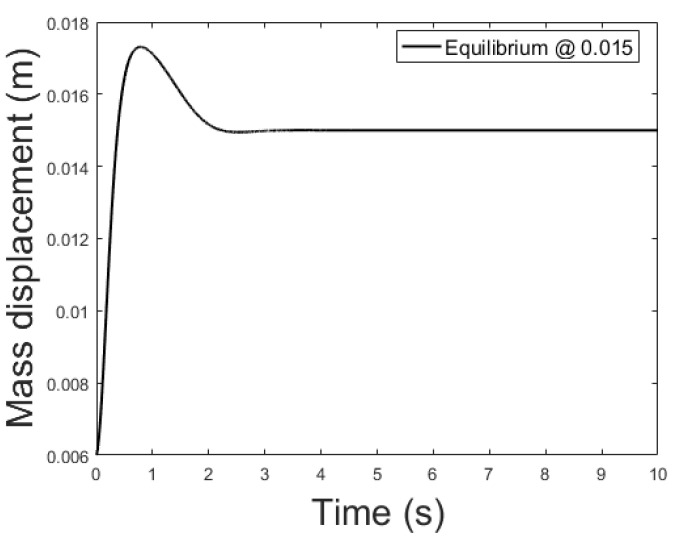
Observer controlled system output with equilibrium at *x* = 0.015.

Figure 14, Figure 15 and Figure 16 depicts the system output, with the observer and the linear controller, for the following initial conditions:

- $x_{0}=0.006$ m, $\hat{x_{0}}=0.002$ m
- $\dot{x_{0}}=0$ m/s, $\hat{\dot{x_{0}}}=0.04$ m/s
- $\psi_{0}=0\;\mathrm{Wb}$, $\hat{\psi_{0}}=0.07\;\mathrm{Wb}$

and equilibrium at 0.005, 0.010 and 0.015 m.

**Figure 17:**
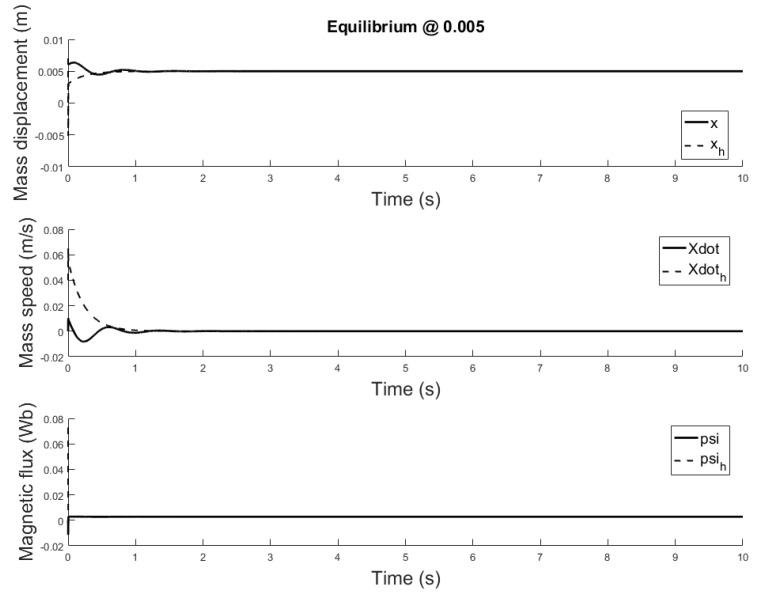
Observer and plant state estimates for initial conditions: $x_{0}=0.006$ m, $\hat{x_{0}}=0.001$ m and $\dot{x_{0}}=0$ m/s, $\hat{x_{0}}=0.04$ m/s and ψ=0 Wb, $\hat{\psi }=0.07\;\mathrm{Wb}$, equilibrium at *x* = 0.005.

**Figure 18:**
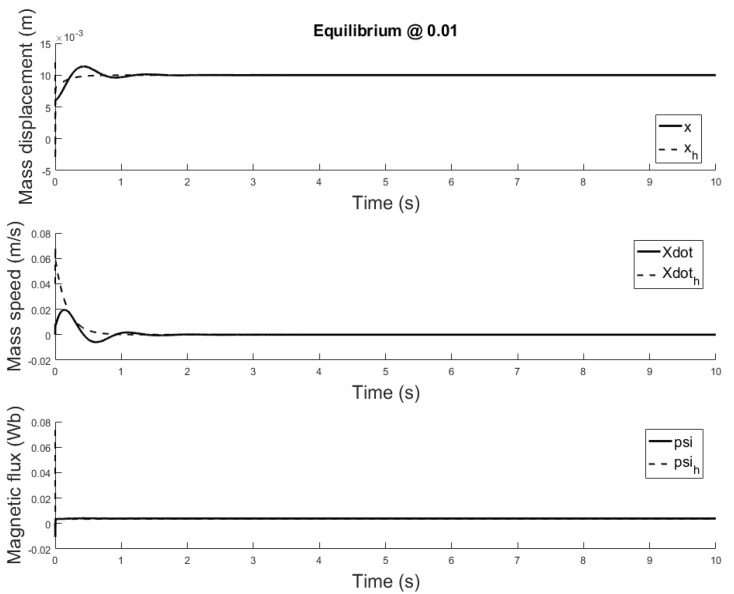
Observer and plant state estimates for initial conditions: $x_{0}=0.006$ m, and $\dot{x_{0}}=0$ m/s, $\hat{x_{0}}=0.04$ m/s and ψ=0 Wb, $\hat{\psi }=0.07\;\mathrm{Wb}$, equilibrium at *x* = 0.01.

**Figure 19:**
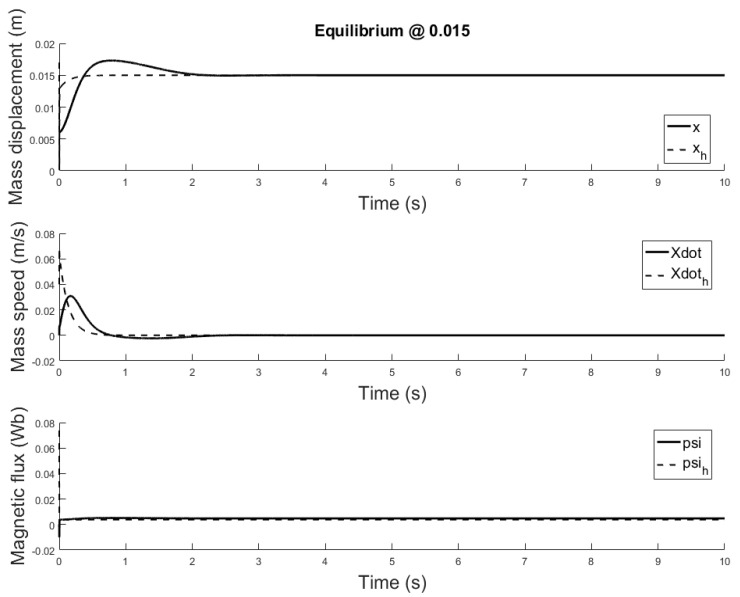
Observer and plant state estimates for initial conditions: $x_{0}=0.006$ m, $\hat{x_{0}}=0.001$ m and $\dot{x_{0}}=0$ m/s, $\hat{x_{0}}=0.04$ m/s and ψ=0 Wb, $\hat{\psi }=0.07\;\mathrm{Wb}$, equilibrium at *x* = 0.015.

The observer is a virtual system, designed to approximate the state variables not available for sensing. A properly designed observer has all initial conditions specified. Despite there isn’t a standard rule, for this study are chosen such that the initial error is equal to e=[0.004,−0.04,−0.07]. Responses of the state variables, for different equilibrium points are depicted in Figure 17, Figure 18 and Figure 19: it is clear that the estimate states converge to the actual state variables while tracking reasonably well the equilibrium value.

## 5. Artificial Neural Network (ANN) Representation of the System

An ANN is an adaptive, often non-linear system, that learns to perform a function (an input/output map) when an input and the corresponding desired or target response set is presented to the untrained ANN [29].

**Figure 20:**
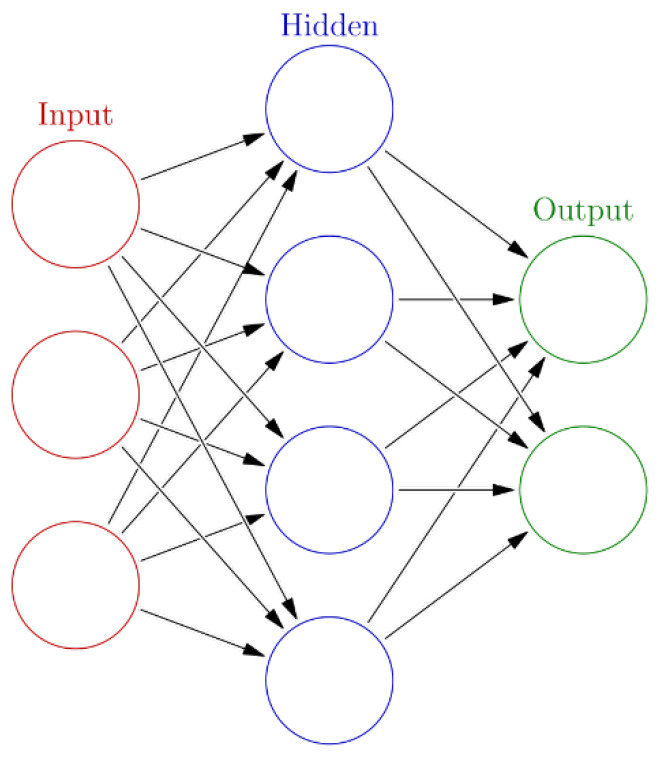
Artificial neural network (ANN) architecture diagram.

An ANN is structured in three layers (Figure 20):

- the input layer, where there is no real processing done, is essentially a “fan-out” layer where the input vector is distributed to the hidden layer;
- the hidden layer, being the computational core of the ANN;
- the output layer, which combines all the “votes” of the hidden layer.

The general mathematical expression that describes an ANN is [29]:

(90) $$y_{ANN}=\left(u{\_}_{n}f_{ANN}\left(x_{ANN}w{\_}_{n}+b_{ANN}\right)\right)+b_{ANN}$$

First, the input $x_{ANN}$ is multiplied by the weight *w__n_*. Second, the weighted input $xw{\_}_{n}$ is added to the scalar bias $b_{ANN}$ to form the net input *n*. The bias is similar to a weight, except that it has a constant input of 1. Finally, the net input is passed through the transfer function $f_{ANN}$, which produces the output $y_{ANN}$.

The equation can be more detailed and further expanded to [29]:

(91) $$h{\_}_{j}=f_{ANN}(\overset{p}{\underset{i=0}{\sum }}w{\_}_{\mathrm{ji}}x_{ANN}{\_}_{i})$$

(92) $$y_{ANN\_k}=\overset{M}{\underset{j=o}{\sum }}(u{\_}_{\mathrm{kj}}h_{\_j})$$

with *p* the number of input nodes, *M* the number of hidden nodes and *K* the number of output nodes.

The activation function $f_{ANN}$ is commonly chosen to be one of the following:

- the logistic sigmoid function (Figure 6), commonly abbreviated as logsig,

(93) $$f_{ANN}\left(z\right)=\frac{1}{1+e^{-z}}$$

- the hyperbolic tangent function (Figure 6), commonly abbreviated as tansig,

(94) $$f_{ANN}\left(z\right)=\frac{1-e^{-2z}}{1+e^{-2z}}$$

The change in error due to output layers weight can be found by Least Mean Square (LSM) algorithms. The output error is given by [29]:

(95) $$E{\_}_{x=}\frac{1}{2}\overset{K}{\underset{k=1}{\sum }}{\left(d{\_}_{k}-y{\_}_{k}\right)}^{2}$$

The back-propagating error corresponding to the hidden layer output can be expressed as the partial derivative [29]:

(96) $$\frac{\partial E{\_}_{x}}{\partial u{\_}_{\mathrm{kj}}}=\frac{\partial E{\_}_{x}}{\partial y{\_}_{k}}\frac{\partial y{\_}_{k}}{\partial u{\_}_{\mathrm{kj}}}$$

Successful training of the network requires calibration of the weights to counteract the error gradient to minimize the output error according to [29]:

(97) $$u{\_}_{\mathrm{kj}}=-\eta \frac{\partial E{\_}_{x}}{\partial u{\_}_{\mathrm{kj}}}=-\eta \delta y{\_}_{k}h{\_}_{j}$$

(98) $$u{\_}_{\mathrm{kj}}^{\mathrm{new}}=u{\_}_{\mathrm{kj}}^{\mathrm{old}}+\Delta u{\_}_{\mathrm{kj}}$$

(99) $$w{\_}_{\mathrm{ji}}=-\mu \frac{\partial E{\_}_{x}}{\partial w{\_}_{\mathrm{ji}}}=-\mu \delta h{\_}_{j}x{\_}_{i}$$

(100) $$w{\_}_{\mathrm{ji}}^{\mathrm{new}}=w{\_}_{\mathrm{ji}}^{\mathrm{old}}+{\Delta w}_{\_\mathrm{ji}}$$

### 5.1. ANN System Configuration

In this section, a feed-forward network organized in layers is proposed to approximate the electro-magneto-mechanical system. The input layer has three neurons, the hidden layer 10 neurons while the output layer has three neurons. The workflow design has the following steps:

- Collect data
- Create the network
- Configure the network
- Initialize the weights and biases
- Train the network
- Validate the network
- Use the network

#### 5.1.1. Generation of ANN Data

The training data is generated using the system described in Section 4. In order to provide some variance to the system, the input current is modelled as:

(101) $$i=i_{0}+I_{0}\cos\left(\omega t\right)\;$$

The training data set for the ANN correspond to the source current (input), the inductance voltage (input), the simulation time (input) and the state variables (output).

#### 5.1.2. ANN Implementation and Training

The Matlab Neural Network Toolbox Mathworks (The MathWorks, Inc.) is used to construct the network. The selected training algorithm is the Levenberg–Marquardt with the following samples set aside for:

- training, 70% of the data;
- validation, 15% of the data;
- and the remaining 15% for testing.

### 5.2. ANN Simulation of the Non-Linear System-Linear Controller-Observer

**Figure 21:**
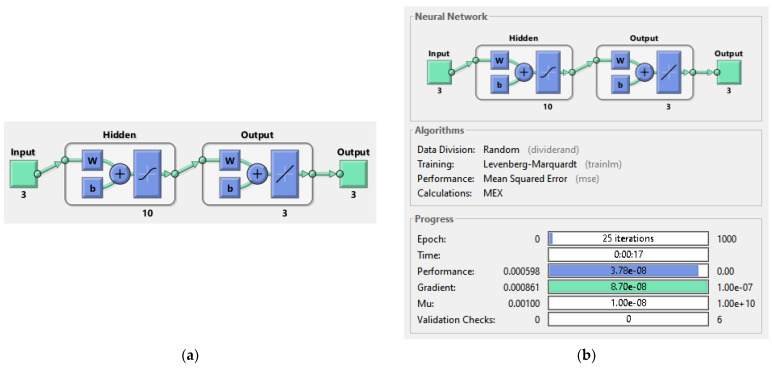
(**a**): ANN architecture setup; (**b**): Matlab Simulink ANN training tool.

**Figure 22:**
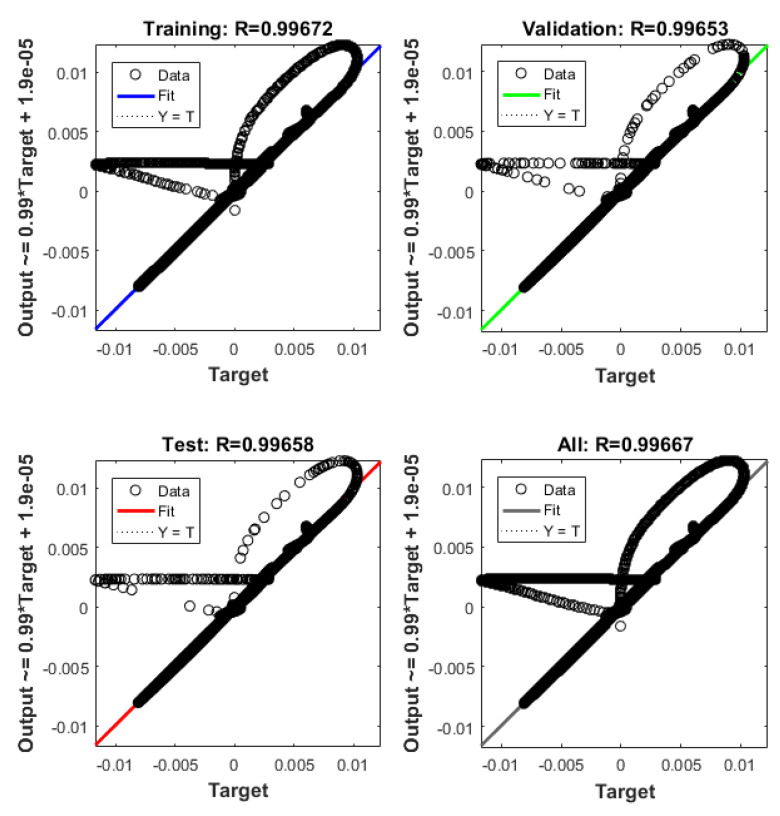
ANN regression plot, equilibrium at *x* = 0.005 m.

**Figure 23:**
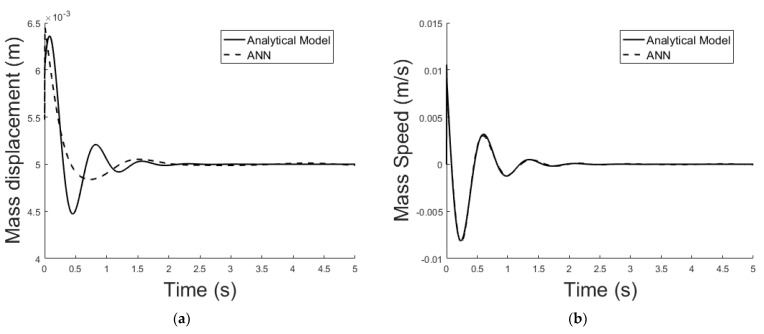
(**a**) Exact model mass displacement versus ANN model mass displacement, equilibrium at *x* = 0.005 m; (**b**) exact model mass speed versus ANN model mass speed, equilibrium at *x* = 0.005 m.

**Figure 24:**
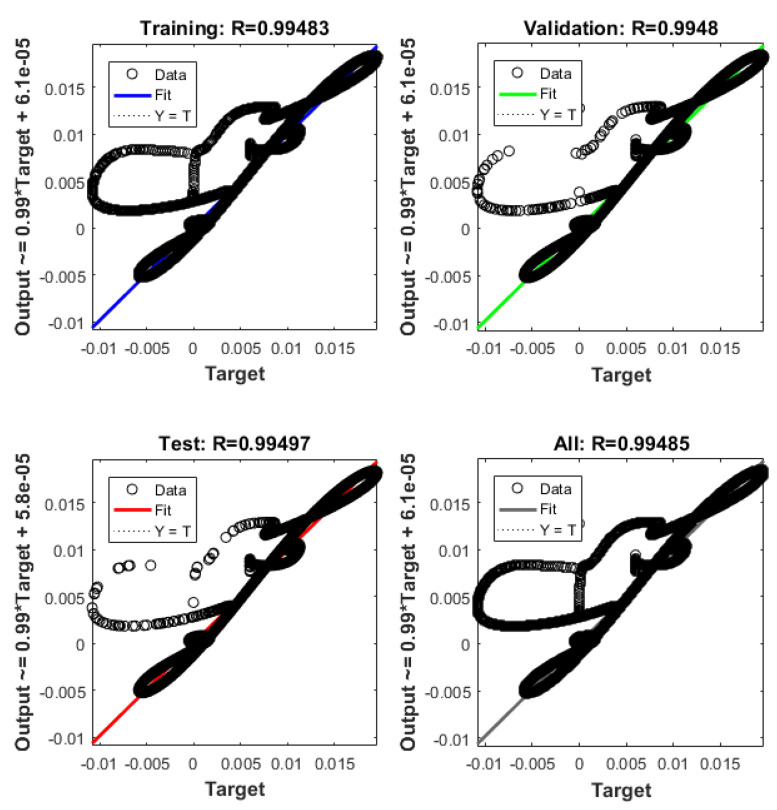
ANN regression plot, equilibrium at *x* = 0.01 m.

**Figure 25:**
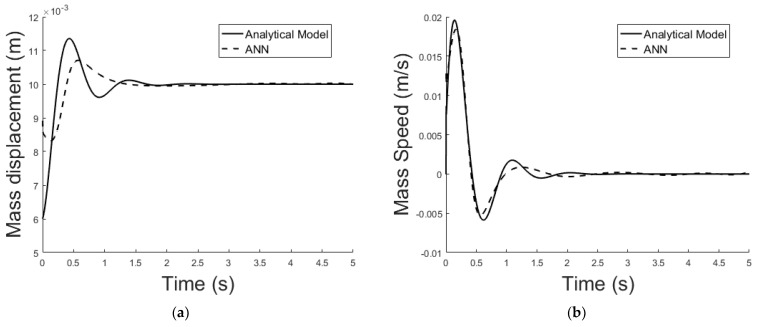
(**a**) Exact model mass displacement versus ANN model mass displacement, equilibrium at *x* = 0.01 m; (**b**) exact model mass speed versus ANN model mass speed, equilibrium at *x* = 0.01 m.

**Figure 26:**
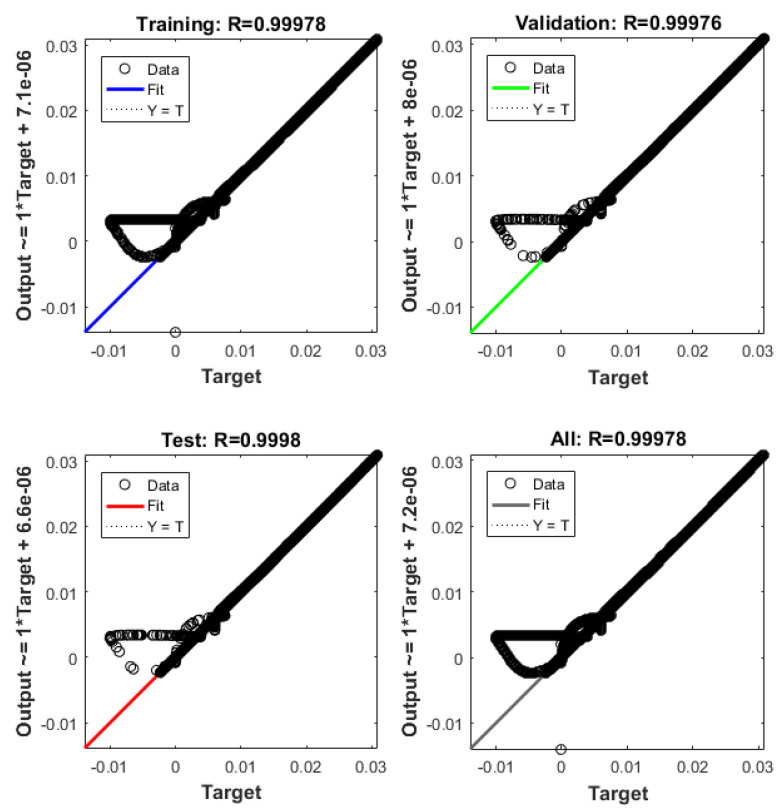
ANN regression plot, equilibrium at *x* = 0.015 m.

**Figure 27:**
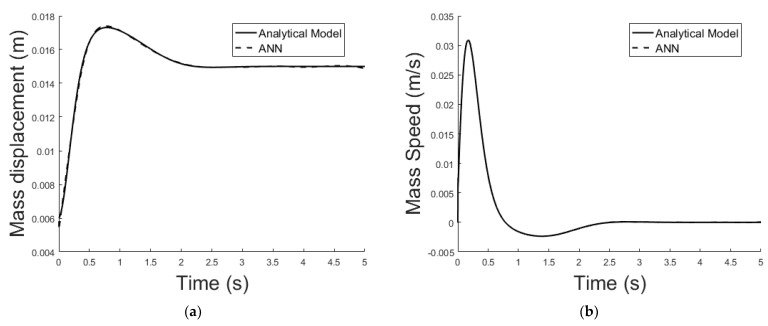
(**a**) Exact model mass displacement versus ANN model mass displacement, equilibrium at *x* = 0.015 m; (**b**) exact model mass speed versus ANN model mass speed, equilibrium at *x* = 0.015 m.

The system described in Section 4.2 is now been simulated by the trained ANN analogue. Figure 21 shows the ANNs setup and the training tool provided by Matlab Simulink. Figure 22, Figure 23, Figure 24, Figure 25, Figure 26 and Figure 27 depict the ANN performance for different equilibrium positions: *x* = 0.005 m, *x* = 0.01 m, *x* = 0.015 m.

The dashed line (Figure 22, Figure 24 and Figure 26) represents the ideal case where the ANN simulated results are an exact replica of the targets. The solid line represents the best-fit linear regression line between trained net outputs and targets, which in our case is adjacent to the ideal case. All cases indicate a good fit, with the scatter manifesting the data points exhibiting a poor fit. R correlates the targets to the simulated net outputs. If R = 1, there is an exact linear relationship between outputs and targets. If R = 0, no linear relationship between outputs and targets can be inferred. All three cases, for different equilibrium points, show a good approximation of the non-linear plant-linear controller-linear observer system output by the ANN (Figure 23, Figure 25 and Figure 27).

## 6. Conclusions

The dynamics of an electro-magneto-mechanical system was investigated in this work. The coupled system simulation was developed and comparatively assessed using different modelling techniques in Matalb/Simulink and Matlab/Simscape. Next, the nonlinear state-space problem formulation and the linearization method using partial derivatives were presented. The resulting linearized system proved both controllable and observable, yet marginally stable. To stabilize and control the system, pole-placement with full-state feedback is employed. To implement the full-state feedback a state observer is introduced and designed. Using the models developed and the simulation results, an ANN equivalent has been configured and tuned for the non-linear plant-linear controller-linear observer system. The model is flexible and accurate; specifically, it is able to adapt to different equilibrium conditions (equilibrium position was varied in the range 0.005 m and 0.015 m) and compensates satisfactorily for changes in current source input (sinusoidal reference input). The system may operate as a contactless displacement sensor or actuator, or in combined mode. In ongoing work, the connection of more than one transducer to the same source will be demonstrated using amplitude modulation. The tuned frequency of each transducer will be defined by assigning different values to the capacitor as shown in Figure 1b. Preliminary analysis shows that such transducer arrays can be used in maritime and aerospace applications, where avoiding physical contact is often a sine qua non requirement, to achieve coordinated sensing and actuation of e.g., space structures like in an orbital station to detect and mitigate the vibrational effects of hits by space debris; or in the maritime, for energy harvesting without violating hull integrity with penetrations to implement physical contact. Other applications are possible too, especially if promising theoretical tools like feedback linearization (instead of the local linearization demonstrated here) are employed.

## Figures and Tables

**Table 1:** Analytical and numerical values for the linearization partial derivatives.

Partial Derivative	Analytical	Numerical
${𝒽}_{x}^{\prime }\left(x_{0},{\dot{x}}_{0},\psi_{0},i_{s0}\right)$	−124.01	−124.86
${𝒽}_{\psi }^{\prime }\left(x_{0},{\dot{x}}_{0},\psi_{0},i_{s0}\right)$	−389.97	−390.76
${𝒽}_{\psi }^{\prime }\left(x_{0},{\dot{x}}_{0},\psi_{0},i_{s0}\right)$	−910.103	−910.45

**Table 2:** State variables, inputs and outputs summary for the linearized system.

States	Input	Output
$\left[\begin{array}{c} \delta x=x-x_{0}\\ \dot{\delta x}=\dot{x}-\dot{x_{0}}\\ \delta i_{L}=i_{L}-i_{L0} \end{array}\right]$	$\delta i=i-i_{0}$	$\delta V_{L}$

## Nomenclature

A	System matrix
A	Cross section (m^2^)
$A_{c}$	Core cross section (m^2^)
$A_{cl}$	Closed loop system matrix
$A_{g}$	Gap cross section (m^2^)
$A_{m}$	Mass cross section (m^2^)
B	Input matrix
B	Magnetic flux density (Wb)
b	Damping constant (Ns/m)
$b_{ANN}$	ANN scalar bias
C	Output matrix
C	Capacitance (F)
D	Feedforward matrix
d	Initial gap length (m)
E	Energy (J)
$E{\_}_{x}$	LSM error
e	Observer error
$\dot{e}$	Observer error variation over time
$F_{em}$	Electromagnetic force (N)
f	Force (N)
𝒻	Function
$f_{ANN}$	Activation function
G	Conductance (Ω^−1^)
𝒽	Function
$I_{0}$	Current amplitude (A)
i	Current (A)
$i_{L}$	Inductance current (A)
$i_{0}$	Equilibrium point current (A)
$i_{s}$	Source current (A)
$i_{s0}$	Source current at equilibrium (A)
K	Kinetic energy (J)
$K_{a}$	Constant
$K_{b}$	Constant
$K_{e}$	Electrical kinetic energy (J)
$K_{gain}$	Gain matrix
$K_{m}$	Mechanical kinetic energy (J)
k	Spring constant (N/m)
L	Inductance (H)
ℒ	Lagrangian quantity
L	Observer gain matrix
$l_{c}$	Core length (m)
$l_{g}$	Gap length (m)
$l_{m}$	Mass length (m)
M	Controllability matrix
m	Mass (Kg)
N	Number of turns
O	Observability matrix
P	Dissipation term (J)
p	Poles
$q_{i}$	Independent variables
R	Resistance (Ω)
ℜ	Magnetic reluctance (H^−1^)
${ℜ}_{c}$	Core magnetic reluctance (H^−1^)
${ℜ}_{g}$	Gap magnetic reluctance (H^−1^)
${ℜ}_{m}$	Mass magnetic reluctance (H^−1^)
s	Constant
T	Potential energy (J)
$T_{e}$	Electrical potential energy (J)
$T_{m}$	Mechanical potential energy (J)
𝒽	Taylor expansion function
$t_{s}$	Stelling time (s)
u	Input control vector
V	Voltage (V)
$V_{L}$	Inductor voltage (V)
$w{\_}_{n}$	ANN weight matrix between hidden and hidden layer
x	Displacement (m)
x	State variable vector
$\hat{x}$	Estimated state variable vector
$x_{ANN}$	ANN input vector
$x_{0}$	Equilibrium point displacement (m)
$\dot{x_{0}}$	Equilibrium point speed (m/s)
$\hat{x_{0}}$	Observer equilibrium point displacement (m)
$\hat{\dot{x_{0}}}$	Observer equilibrium point speed (m/s)
y	Output vector
$\hat{y}$	Estimated output vector
$y_{ANN}$	ANN output
z	Neutral position of electromagnetic system
δi	Current deviation from equilibrium point (A)
δx	Displacement deviation from equilibrium point (m)
$\delta \dot{x}$	Speed deviation from equilibrium point (m/s)
$\delta \ddot{x}$	Acceleration deviation from equilibrium point (m^2^/s)
δψ	Magnetic flux deviation from equilibrium point (Wb)
$\delta \dot{\psi }$	Voltage deviation from equilibrium point (V)
ζ	Damping ratio
λ	Eigenvalue
$\mu_{c}$	Core relative material permeability
$\mu_{m}$	Mass relative material permeability
$\mu_{0}$	Air permeability (H/m)
$\xi_{0}$	Inductance constant
$\xi_{1}$	Inductance constant
$\xi_{2}$	Inductance constant
ψ	Magnetic flux (Wb)
$\psi_{0}$	Magnetic flux at equilibrium (Wb)
$\hat{\psi_{0}}$	Observer equilibrium point magnetic flux (Wb)
ω	Natural frequency
